# Immunological Aspects of *Candida* and *Aspergillus* Systemic Fungal Infections

**DOI:** 10.1155/2013/102934

**Published:** 2013-01-21

**Authors:** Christoph Mueller-Loebnitz, Helmut Ostermann, Anke Franzke, Juergen Loeffler, Lutz Uharek, Max Topp, Hermann Einsele

**Affiliations:** ^1^Medical Writer, Goldammerweg 4, 91301 Forchheim, Germany; ^2^University Hospital of Munich-Großhadern, 81377 Munich, Germany; ^3^Department of Hematology, Hemostaseology and Stem Cell Transplantation, University of Hannover, 30625 Hannover, Germany; ^4^Medical Clinic II, University of Würzburg, 97080 Würzburg, Germany; ^5^Department of Hematology, Charité, Campus Benjamin Franklin, 12203 Berlin, Germany

## Abstract

Patients with allogeneic stem cell transplantation (SCT) have a high risk of invasive fungal infections (IFIs) even after neutrophil regeneration. Immunological aspects might play a very important role in the IFI development in these patients. Some data are available supporting the identification of high-risk patients with IFI for example patients receiving stem cells from TLR4 haplotype S4 positive donors. Key defense mechanisms against IFI include the activation of neutrophils, the phagocytosis of germinating conidia by dendritic cells, and the fight of the cells of the innate immunity such as monocytes and natural killer cells against germlings and hyphae. Furthermore, immunosuppressive drugs interact with immune effector cells influencing the specific fungal immune defense and antimycotic drugs might interact with immune response. Based on the current knowledge on immunological mechanism in *Aspergillus fumigatus*, the first approaches of an immunotherapy using human T cells are in development. This might be an option for the future of aspergillosis patients having a poor prognosis with conventional treatment.

## 1. Introduction

Invasive fungal infections (IFIs) have a great impact on clinical course and outcome. Of 100 patients with acute myeloid leukemia (AML), 7% have proven, 7% probable, and 43% possible IFI. About half of patients developing proven IFI do not survive.

Criteria for IFI diagnosis defined by EORTC and MSG include recent history of neutropenia (<0.5 × 10^9^ neutrophils/L for >10 days) temporally related to the onset of fungal disease and receipt of allogeneic stem cell transplantation (SCT), as well as inherited severe immunodeficiency such as chronic granulomatous disease or severe combined immunodeficiency [1]. In addition, EORTC/MSG mentioned diagnostic criteria such as prolonged use of corticosteroids at a mean minimum dose of 0.3 mg/kg/day of prednisone equivalent for >3 weeks and treatment with T cell immunosuppressants, for example, cyclosporine or TNF-*α* blockers, specific monoclonal antibodies (e.g., alemtuzumab), or nucleoside analogues during the past 90 days [1]. The size of the hospital seems to be also associated with the risk of developing a fungal infection, probably due to higher numbers of patients at risk being treated in bigger hospitals [2].

Immunological aspects play an important role in the development of invasive fungal infection, especially in the development of invasive aspergillosis. To discuss the importance of the different cells of the immune system for the development of IFI, a special symposium was held in Würzburg, Germany. This paper summarizes the results of the symposium.

## 2. Clinical Aspects 

About 60% of patients with IFI suffer from hematological neoplasia, 20% have chronic lung diseases, and most of the remaining patients affected have solid organ transplantation or systemic diseases such as rheumatism [3]. Nearly all IFI patients have been pretreated with corticosteroids or antibiotics pointing at additional infectious problems. In nonhematological patients, *Aspergillus* infections are less frequent. However, Belgian authors registered 69 *Aspergillus *infections in a series of 1850 ICU patients—mostly in patients with COPD but also with rheumatic diseases and liver cirrhosis [4]. Neutropenia is the most important established risk factor. Portugal et al. used an index, based on the area above the curve (area between 500 neutrophils and the curve multiplied by the days (D-index)) [5]. The index predicted mold infections in high-risk neutropenic patients only with a low specificity, but the negative predictive value was high, and IFI could be excluded with a high probability in some patients. However, the absolute number of patients in which invasive aspergillosis could be excluded by Portugal et al. was very low.

As aspergillosis is an infectious disease transmitted by inhalation, air contamination might be a risk factor. Nevertheless, there is no real evidence for the preventive efficacy of air filtration in SCT units or facemasks. Rupp et al. [6] measured *Aspergillus* air concentrations between 1992 and 1999 in a transplant unit repeatedly and classified the results in three categories: >15 CFU/m^3^ (*n* = 19: group A), 5–15 colony forming units (CFU)/m^3^ (*n* = 28; group B), and no *Aspergillus* detection (*n* = 100; group C). The proportion of periods in which a case of invasive aspergillosis (IA) was diagnosed during the 14 days immediately following a test result was 17% (group A), 15% (group B), and 13% (group C). After 30 days, these percentages increased to 30% in group A, 24% in group B, and 19% in group C. The differences were not significant, just a trend was observed (*P* = 0.29).

A meta-analysis evaluating studies investigating the impact of high efficiency particulate airfilter (HEPA) or laminar air flow (LAF)—filtration did not show a lethality reduction [7]. The IFI rate was numerically lower, but this subgroup analysis was not sufficiently powered. A prospective randomised study investigating the use of facemasks for IA prevention in high-risk patients also did not show an advantage of this reverse isolation approach [8]. During followup, even more fungal infections were observed in patients wearing masks. However, some impact might be possible: a Portuguese study measuring *Aspergillus* concentrations before and after renovation of a hematology unit detected more infections before renovation [9].

The control of the underlying disease is very important for IFI outcome. Patients with uncontrolled disease are more immunocompromised than patients recovering from leukemia. Noncontrolled underlying disease is one of the strongest risk factor for an unfavorable outcome [10].

## 3. Genetic Risk Factors for Invasive Aspergillosis

IFI manifestation and severity depends on three factors: the intensity of the exposition with fungal conidia, the degree of immunosuppression, and the efficacy of the immunological defense mechanisms. The target structures on fungal pathogens, the pathogen-associated molecular patterns (PAMPs), are highly conserved molecular cell wall structures. PAMPs are recognized by pattern recognition receptors such as TLRs (toll-like receptors) and non-TLRs such as dectin-1 expressed on phagocytes and dendritic cells (DCs). It is supposed that TLRs play a key role in the initiation of the fungal-specific immune response. The activity of the TLRs depends on fungal species, morphology (hyphae or spores), and the infected area. TLR signaling is performed via the adaptor protein MYD88, which initiates the production of proinflammatory cytokines.

The largest study analyzing the role of TLR polymorphism has been conducted at the Fred Hutchinson Cancer Center, Seattle, WA, USA. About 20 SNPs (single nucleotide polymorphisms) for TLR2, TLR3, TLR4, and TLR9 have been characterized in 336 unrelated allogeneic SCT donor/recipient pairs followed by a validation study comparing 103 donor/recipient pairs (IA cases) with 263 matched donor/recipient pairs not developing aspergillosis (controls) [11]. The study identified as independent risk factors for the development of an IA the donor TLR4 haplotype S4, CMV seropositivity and acute GvHD—but only in unrelated donor/recipient pairs. The haplotype being associated with reduced function occurs in about 6% of Caucasians. Only one of 83 allogeneic SCT recipients without risk factor (S4− plus CMV−) developed IA within 3 years. The highest IA incidence was observed in donor/patients pairs being S4+ (donor) plus CMV+ (recipient) (21.7%; 5 of 23 patients). In the largest group (S4− plus CMV+; *n* = 136), 10% of patients developed IFI. It can be concluded that the identification of a high-risk group is not important, but the clear detection of a low risk group with an incidence near to zero.

A retrospective analysis observed a significant association between TLR1 and TLR6 polymorphisms of the recipient and IPA incidence in 127 patients with allogeneic SCT [12]. However, monogenetic mechanisms probably do not explain completely the association due to the multiple detection ways of the pattern recognition receptors.

### 3.1. Non-TLRs as Initiators of Fungal Specific Immune Responses

The most important non-TLR receptor is dectin-1, a transmembrane signaling-receptor expressed especially in the lung and the GI-tract on DCs, phagocytic cells, B cells, eosinophils and T cell subsets. The dectin-1-expression, which is important for the detection of *Candida albicans* and *A. fumigatus*, is reduced by steroids. Dectin-1 acts via the CARD9 pathway similarly to the adaptive immunity resulting in the production of IL-23 and the formation of Th17 cells. The induced inflammation process is important for the recruitment of neutrophils combating the fungal infection but possibly also resulting in collateral damage due to excessive immune reactions. Dectin-1 promotes via NFkappaB the release of proinflammatory cytokines such as TNF*α*. In a murine model, dectin-1 deficiency was associated with a predisposition for *Candida* infections. In an index family, a homozygote CARD9 loss-of-function mutation with loss of dectin-1 triggered TNF*α* production resulted in mucocutaneous candidiasis [13]. After retroviral transduction of human CARD9 wild type to mutated human CARD9, a loss of function mutation with a reduction of Th17 cells due to lacking proinflammatory cytokines was observed in mice [13]. Polymorphisms within the dectin-1 pathway have not been distinguished so far, which might be associated with a higher risk of developing *Candida* infections. 

### 3.2. IL-10 and Invasive Aspergillosis

IL-10 is an immunoregulatory cytokine counterbalancing the antifungal immune response in multiple ways. It inhibits the development of DCs, downregulates MHC class II, and induces regulatory T cells (Tregs). The recruitment of Tregs to the infection site supports fungal immune evasion mechanisms by forming a barrier inhibiting the antifungal T cell response. 

An IL-10 promoter analysis identified several SNPs correlating with IL-10 production. The IL-10 promoter ACC haplotype is associated with lower IL-10 production and reduced IA risk in patients after allogeneic SCT [14]. During the first 2 years after allogeneic SCT, 7% of patients with IL-10 promoter ACC haplotype developed IA compared to 19.7% with non-ACC haplotype. In models with IL-10 treated human macrophages, superoxide and cytokine production was reduced together with lower *Aspergillus* destruction rates [15]. IL-10-/- knockout mice showed enhanced antifungal Th1 immune responses after *Aspergillus* infection associated with lower mortality. Increased IL-10 serum levels correlate with poor prognosis in nonneutropenic IA patients [16].

IL-10 has immunoregulatory properties in an inflammatory microenvironment by differentiating naive CD4^+^ cell to Tregs. Tregs are important for the immune homeostasis as well as for induction and maintenance of peripheral tolerance. They suppress autoimmunity, tumor immunity, allergy, and infections including the suppression of the antifungal immune response. Whether Tregs cause pathogen resistance or have protective effects depends on the balance between effector T cells and Tregs. The balance is necessary for pathogen clearance and limitation of inflammation. Tregs are needed to lower immune response and to avoid collateral damage. However, if Tregs outnumber effector T cells, antifungal Th1 response is suppressed and the pathogen persists. If the effector cells dominate, we have pathogen clearance together with an organ destructing inflammatory reaction. A murine model demonstrating reduced fungal growth but also enhanced organ destructing inflammation after Treg reduction confirmed this observation [17].

## 4. Neutrophils

Neutropenia is among the most important risk factors for IA, but IA also occurs in nonneutropenic patients, and some clinical observations even suggest that the proportion of IA cases in nonneutropenic patients is increasing. Some other fungal infections, for example, by *Pneumocystis jiroveci* or *Cryptococcus neoformans* are associated with T cell defects and much less influenced by neutrophils counts or function. Nevertheless, neutrophils contribute considerably to antifungal immunity. They are the most efficient cell type with regard to fungal killing, determine, for example, the transcriptional adaptation of *C. albicans* in the blood, and inhibit filamentation. As a first line immune defense, neutrophils recognize fungi rapidly and efficiently (innate recognition).

Neutrophils can directly and indirectly detect fungi by a set of receptors including TLR2, TLR4, dectin-1, several Fc receptors, the complement receptors CR1, CR3 (fungi bind complement factors and are subsequently detected by CRs but may also bind directly to these receptors), and perhaps CEACAM which—although direct fungal binding has not been shown—may interact with TLRs and change their signaling. Neutrophils are highly motile, activated very rapidly, and selectively attack *C. albicans *hyphae and wrap them within one hour, whereas they seem to ignore yeast forms. Indeed, in the case of *C. albicans*, neutrophils are capable of initiating a morphotype-specific activation program in response to invasive filamentous forms. In this case, the activation of neutrophils depends on the MAP-kinase signaling, especially the ERK signaling pathway as inhibition of the ERK signaling pathway abolishes *Candida albicans* induced neutrophil migration [18]. Neutrophils are very rapidly activated by these phosphorylation events. *In vitro*, neutrophils reduced *Candida albicans* hyphal growth by 80% compared to controls and killed 50% of *C. albicans* hyphae within 3 hrs—observations that further underline the important role of neutrophils in antifungal immunity.

The microbicidal mechanisms of neutrophils include oxidative burst, NET formation, and release of antimicrobial substances. In mammals, only neutrophils are able to “liquefy any part of the body” including connective tissue [19]. Neutrophils exert many microbicidal mechanisms including the oxidative burst characterized by production of oxygen radicals. Fungi such as *Candida* induce reactive oxidative intermediates (ROIs) probably through components of their cell walls. However, the paradigm of the oxidative burst being essential for *Aspergillus* killing has been challenged recently.

An investigation comparing an *Aspergillus* mutant with *Aspergillus *wild type demonstrated a considerably higher sensitivity to hydrogen peroxide and menadione, two oxygen radical donors, of the mutant [20]. The wild type induces catalases after contact with oxygen radicals, whereas the mutant is incapable of doing so. However, the oxygen radical sensitive mutant did not show a significant loss of virulence in a murine infection model compared to the wild type. Furthermore, the inhibition of oxygen radical formation in human neutrophils had no significant effect on *Aspergillus* killing [20]. 

Which other neutrophil mechanisms can contribute to *Candida* and *Aspergillus* elimination? NET formation is another possibility. Neutrophil extracellular traps capture and kill *Candida albicans* yeast and hyphal forms [21]. A recent case report suggested control of aspergillosis in a patient with chronic granulomatous disease (CGD) by gene therapeutic restoration of NET formation [22]. However, several other host factors might be important too: factor H and factor H-released protein 1 bind to human neutrophils via complement receptor 3, mediate attachment to *C. albicans*, and enhance neutrophil antimicrobial activity [23]. Human epithelial cells establish direct antifungal defense through TLR4-mediated signaling [24]. The exact role of neutrophils in this complicated context is still not fully understood.

## 5. Monocytes/Macrophages/Dendritic Cells 

Every human inhales 10,000 to 15,000 liters of air daily including 50–500 *Aspergillus* spores. Only 10% of spores are *A. fumigatus: A. niger* or *A. versicolor* occur more frequently. After inhalation, the spores enter the humid and warm environment of the alveoli.

Alveolar macrophages constitute the first line of host defense against *A. fumigatus *invasion. In immunocompromised patients, the conidia germinate, germlings develop and enter the interstitial space where DCs phagocyte the germinating conidia. If the infection further develops, the hyphae enter the capillary lumen and blood vessels. There, the cells of the innate immunity, PMN, monocytes, and NK cells fight against germlings and hyphae. 

Contrary to most bacteria, pathogenic fungi change their morphology considerably during the early infectious period resulting in different fungal surface structures. *Aspergillus* has four morphologies: dormant conidia, swollen conidia, germlings, and hyphae. Little is known about interactions between dormant conidia and innate immunity. Alveolar macrophages and neutrophils are able to recognize and phagocyte resting conidia but probably cannot kill them. For killing, conidia swelling is necessary. Only swollen conidia are detected by receptors (dectin-1, TLR2, and TLR4 in DC and also DC-SIGN) and intracellularly killed by alveolar macrophages and neutrophils. 

Resting conidia are covered by hydrophobin layers, such as rodA [25]. In a model, dormant conidia of *rodA* mutant (no *rodA* in the cell wall) were detected by the immunity and induced DC maturation and activation as wild type germlings and hyphae do. The *rodA* mutant provoked heavy cytokine and chemokine release (IL-6, IL-8, and IL-12) similar to wild type germ tubes or hyphae, whereas nearly no induction was seen with the wild type conidia. It can be concluded that rodA is the factor enabling conidia to avoid detection by the innate immunity.

90% of swollen spores are killed within 30 h by alveolar macrophages [26]. In the phagolysosomal compartment, the dormant spores become active and swell enabling macrophages to kill them [27]. Alveolar macrophages can destroy conidia via nonoxidative mechanisms; rabbit alveolar macrophages killed *Aspergillus* conidia in an anaerobic environment. Dexamethasone inhibits *A. fumigatus* killing by alveolar macrophages [28]. GM-CSF prevents dexamethasone suppression of killing of *Aspergillus fumigatus* conidia and restores the killing ability of macrophages.

### 5.1. Monocytes

Monocytes (3.8% of leukocytes) are macrophage and DC precursors. They destroy foreign structures by phagocytosis and innate immunity activation via antigen presentation. Swollen *Aspergillus *conidia are efficiently phagocyted and intracellularly killed [29]. The killing can be enhanced by granulocyte-macrophage colony-stimulating factor (GM-CSF) [30] or IFN-*γ* [31]. Monocytes increase the expression of CCL20, which shows activity towards neutrophils, monocytes, and naive T lymphocytes and mobilizes intracellular calcium. Increased monocyte ICAM-1 expression augments the infiltration of neutrophils and mast cells [29]. 

### 5.2. Dendritic Cells—The Bridge between Innate and Adaptive Immunity

DCs activate the innate immunity by cytokine and chemokine release and can present antigens for T cell activation. They internalize both *Aspergillus* conidia and hyphae and transport them from the airways to draining lymph nodes [32]. DCs ingest conidia through coiling phagocytosis, probably involving DC-SIGN and complement receptor (CR) 3, leading to the priming of Th1 responses [32]. Hyphal phagocytosis by DCs results in IL-4 and IL-10 production* in vitro* [32].

Immature DCs recognize microbial structures such as *β*-glucan via dectin-1 receptor or other fungal structures via TLR2 and TLR4. If the DC matures, the form changes from round to stretched and cytokines such as TNF*α*, IL-12p70, and IL-10 are released, T cells polarized (Th1, Th2, Th17, Tregs), and NK cells activated via IFN-*γ* and IL-2 release.

DCs interact with *A. fumigatus* via several immune receptors such PTX3 and TLR [33]. Different pro- and anti-inflammatory cytokines are upregulated. The expression of CCL20, a cytokine with strong proinflammatory and chemotactic properties for granulocytes and T cells, is 10,000-fold increased. PTX3 also increases considerably within 3 hours. Dectin-1 interacts efficiently with *A. fumigatus*. Dectin-1 knockdown by siRNA leads to reduced TNF*α* expression (50% reduced gene expression, 60% less protein) and similar decreases of IL-12 and IL1-*β*.

Immunomodulatory agents such as cyclosporine A, tacrolimus, and rapamycin have potent antifungal activity, most notably against *C. neoformans*, *Candida *spp., and *Aspergillus *spp. The targets of their antifungal activity are fungal homologs of mTOR. Mycophenolate and rapamycin decrease the secretion of proinflammatory cytokines in human DCs after stimulation with *A. fumigatus* germlings markedly [33]. The TNF*α* and IL-12 gene expressions were reduced by 52% and 94%, respectively.

## 6. Immunotherapeutic Approaches Using T Cells

After allogeneic SCT, the neutrophils regenerate, but Th1 immune response defects often persist. The most important study on T cells and early aspergillosis investigated the transfer of functional immune responses to pathogens (specific T cells against *A. fumigatus*) after haploid SCT [34]. The included haploidentical recipients with evidence of aspergillosis had marked immune defects with delayed T cell reconstitution. The treatment significantly reduced *Aspergillus* galactomannan antigenemia. In controls, galactomannan antigenemia remained positive for fifteen weeks.

In an *in vitro* experiment, highly purified and functionally active human Th1 cells against *A. fumigatus* were generated from donor PBMCs by stimulation with *A. fumigatus* extract [35]. The Th1 cell lines secreted IFN-*γ* and IL-2 and significantly increased hyphal damage by neutrophils [36]. In a second project, activation proteins were manufactured in a recombinant eukaryotic expression system (expression vector with FLAG-tagged recombinant protein), and the cytoplasmic expression was performed in human cells to circumvent contamination with endotoxin or yeast protein [36]. It followed the activation of transgenic expression by sodium butyrate and the purification of the FLAG-tagged proteins with antibody-conjugated agarose.

Two proteins were selected for the investigations, Asp f16 and Asp f1. Asp f16 induces protective Th1 responses in mice when given together with an adjuvant [37] and the proliferation of human PBMCs [38]. Asp f1 induces Th2-based allergic responses in mice [39] and humans [40]. Protein production was difficult due to problems with point mutations. De novo immune responses to recombinant proteins were not detected during the second project. However, restimulations for one week resulted in T cell responses especially against Asp f16 (Th1 directed). Responses against Asp f1 were observed only occasionally and were predominantly allergic, Th2 directed [36].

The third step was the determination of antigenic epitopes and their MHC restrictions [36]. Only CD4^+^ but not CD8^+^ T cells of healthy donors showed a response to Asp f16. The MHC class II response might be the typical reaction, and no MHC class I response against *Aspergillus* exists.

During search for immunodominant epitopes, several Asp f16 epitopes were evaluated, and five candidates coding different proteins including FHT protein were further investigated [36]. Asp f16-specific cell lines from 3 different HLA-DRB1*01 positive donors were restimulated with five synthetic peptides. One candidate, the FHT protein, induced a response (release of IFN-*γ*) in all of the three cell lines. An Asp f16-specific cell line of one HLA-DRB1*01 positive donor generated with recombinant protein responded to rechallenge with Asp f16 protein and FHT peptide. Asp f16-specific T cell clones recognized their target antigen (*Aspergillus *antigen or FHT peptide) on DCs incubated together with viable fungus (*Candida albicans* and *A. fumigatus*). Viable *Aspergillus* restimulated Asp f16-specific FHT T cell clones (IFN-*γ* release) but did not stimulate a CMV-specific T cell clone. This means that antigen presented via MHC class II, vital conidia, or control antibodies specifically activates Aspf16-specific T cell clones. However, MHC class II blocking antibodies inhibited the activation. Fungal morphology is also important: Asp f16-specific T cell clones are activated by germinating conidia and hyphae, whereas resting conidia do not induce activation.

Activated Asp f16-specific T cell clones enhanced monocyte-mediated fungal killing [36]. The monocytes were preincubated with supernatant (SN) taken from Asp f16-specific T cell clones activated by DC pulsed with peptide or fungal antigen. In pulsed SN, fungal killing by monocytes increased compared with control supernatant. Activated T cells showed markedly improved phagocytosis ability: the transfer of specific T cell populations might enable an improved immunological control in high-risk patients.

Memory Th1 cells specific for an Asp f16 epitope activated and expanded with synthetic peptide might be able to boost antifungal innate immunity by enhancing the fungicidal activity of monocytes [36]. The next investigational steps will be the characterization of immune responses to further recombinant proteins of* A. fumigatus*, followed by the determination of the frequency of antigen-specific memory cells in healthy individuals and patients suffering from or surviving invasive aspergillosis. The detection of antigenic proteins by fractionation of cellular *A. fumigatus* extracts, the cloning of T cell receptors of *Aspergillus*-specific T cells, and the transfer to recipient T cells are also planned [36].

## 7. Natural Killer Cells in Fungal Defense

Natural killer (NK) cells express Fc3 receptor, an immunoglobulin receptor being theoretically able to interact with the humoral defense against fungal infections. After transplantation, NK cells can damage antigen-presenting cells possibly avoiding GvHD. NK cells destroy recipients' T cells inhibiting graft failure, improve engraftment, and prevent relapses by killing tumor cells. The available evidence is restricted to experimental data. Ruggeri et al. [41] improved the chimerism with increasing doses of NK cells in animals, and Asai et al. [42] inhibited GvHD in animals and increased survival rates. In older investigations, the recurrence rate in mice was reduced [43].

A literature research yielded 7-8 publications about NK cells and *Aspergillus*. In *Aspergillus* infections, chemokines attract NK, and the lung concentration of NK cells increases. IFN-*γ* production of NK cells seems to be an important defense mechanism as IFN-*γ* promotes the migration of other effector cells to the infected region and IFN-*γ* knockout mice are sensitive to aspergillosis. 

Park et al. [44] investigated the role of NK cells in C57 mice infected transtracheally with *A. fumigatus* after neutrophil depletion by antibodies. In *Aspergillus* infected mice, predominantly NK cells produced IFN-*γ*. NK depletion resulted in significantly reduced IFN-*γ* levels in the lung. Additionally, the survival of granulocyte-depleted animals was better compared with granulocyte plus NK cell depleted animals. A comparison between wild type mice and IFN-*γ* knockout mice confirmed the protective NK cell effect mediated by IFN-*γ*.

The incubation of wild type mice macrophages together with NK cells did not result in macrophage activation by NK cells. However, there was a delayed effect on fungal growth: macrophages inhibited fungal growth very little, but together with NK cells, the inhibition increased to 50%. IFN-*γ* knock out NK cells had markedly smaller effects.

A clinical study included 30 patients after haploidentical transplantation with highly purified NK cells [43]. About one-third of patients died of pneumonia, one-third of them of fungal pneumonia. This is a relatively low infection rate for these highly pretreated patients. In comparison to matched patients from the EMBT database, mortality and infection rates were reduced. In aspergillosis during neutropenia, chemokines cause NK cell infiltration in the infected area, and NK cells are able to activate immune mechanisms and possibly enhance fungal T cell response. It can be concluded that NK cell might offer an important benefit for the infection defense.

## 8. Conclusions

The determination of genetic factors is an interesting approach for the identification of patients with a high IFI risk. We are just beginning to understand the role of the different immune cells such as neutrophils, monocytes, macrophages, DCs, T cells, and natural killer cells in the defense against IA and other fungal infections. Early studies investigating the immune cell transfer were promising, but results need to be confirmed by prospective randomized studies. The first attractive application of immunomodulating to treat invasive fungal infections is the treatment with certain cytokines, for example, IFN*γ*/GM-CSF to improve antigen presentation and thus to enhance immune defense against fungal pathogens. Especially in hepatolienal candidiasis and refractory IA, the addition of GM-CSF/IFN*γ* was shown in some case series to improve outcome.
